# Comparison of Plant Morphology, Yield and Nutritional Quality of *Fagopyrum esculentum* and *Fagopyrum tataricum* Grown under Field Conditions in Belgium

**DOI:** 10.3390/plants10020258

**Published:** 2021-01-28

**Authors:** Lauranne Aubert, Christian Decamps, Guillaume Jacquemin, Muriel Quinet

**Affiliations:** 1 Earth and Life Institute-Agronomy, Université Catholique de Louvain, B-1348 Louvain-la-Neuve, Belgium; lauranne.aubert@uclouvain.be (L.A.); christian.decamps@uclouvain.be (C.D.); 2 Unité Productions Végétales, Département Productions Agricoles, Centre Wallon de Recherches Agronomiques, B-5030 Gembloux, Belgium; g.jacquemin@cra.wallonie.be

**Keywords:** common buckweat, Tartary buckweat, proteins, amino acids, minerals, yield, seed

## Abstract

Buckwheat is a pseudocereal with high nutritional and nutraceutical properties. Although common buckwheat (*Fagopyrum esculentum*) is the main cultivated species, Tartary buckwheat (*Fagopyrum tataricum*) is gaining interest. In this paper, we compared plant growth, yield-related parameters and seed nutritional qualities of two varieties of *F. esculentum* and *F. tataricum* under field conditions in Belgium. *Fagopyrum esculentum* flowered earlier, produced less nodes, less branches, less inflorescences, but more flowers per inflorescence than *F. tataricum*. The yield was higher in *F. tataricum*, while the thousand-grain weight was higher in *F. esculentum*. Yield ranged between 2037 kg/ha and 3667 kg/ha depending on the species and year. Regarding nutritional qualities, seeds of *F. esculentum* contained more proteins (15.4% vs. 12.8%) than seeds of *F. tataricum* although their amino acid profile was similar. Seeds of *F. esculentum* contained also more Mg (1.36 vs. 1.15 mg/g dry weight (DW)) and less Fe (22.9 vs. 32.6 µg/g DW) and Zn (19.6 vs. 24.5 µg/g DW) than *F. tataricum*. The main difference between seed nutritional quality was the concentration of flavonoids that was 60 times higher in *F. tataricum* than in *F. esculentum*. Both species grow well under Belgian conditions and showed good seed quality.

## 1. Introduction

Buckwheat is a dicotyledon belonging to the Polygonaceae family and the *Fagopyrum* genus [1]. Inside this genus, two species are cultivated, *Fagopyrum esculentum* Moench (common buckwheat) and *Fagopyrum tataricum* (L.) Gaertn (Tartary buckwheat) [2]. The former accounts for 90% of the world buckwheat production. Both species originated from China [3,4]. *Fagopyrum esculentum* may have been cultivated for as long as 6000 years, and the Yunnan region of China has been shown to be a significant site of domestication of common buckwheat [3]. From southern China, common buckwheat was introduced to Asian countries through two main routes, via Himalayan region and Tibet or via northern China and Japan [4,5]. It has been suggested that buckwheat was introduced in Europe during the period 4000–2800 BP but it did not become a popular crop until the Late Medieval period [3,4,6]. From the 17th century, buckwheat was introduced to America and South Africa by emigrants [3,4]. *Fagopyrum tataricum* originates from the Himalayan region and has been cultivated for at least 4000 years [7]. It grows wild in China, Siberia, Tibet, Kashmir and northern Pakistan [3] and two independent domestication events occurred in southwestern and northern China [7]. It was introduced to other countries from northern China [7]. Buckwheat is considered as a pseudocereal, such as amaranth (*Amaranthus* spp.) and quinoa (*Chenopodium quinoa* Willd) [3,8]. Pseudocereals are dicotyledonous crops that are not related to the cereals; however, their seeds do share similarities with cereal grains [9]. Their seeds have similar physical appearance and contain high starch content similar to true cereals [10]. However, the anatomy of the pseudocereal grains differs from the staple cereals in that they contain less endosperm and a higher proportion of embryos [8]. Pseudocereal grains have also high nutritional and nutraceutical qualities; however, despite their potential advantages, their agronomic potential remains poorly characterized [8,10,11]. Buckwheat is widely distributed in the world but grows for the most part in the northern hemisphere [9]. The largest producing countries of buckwheat are China, Russian Federation, Ukraine and France [12]. The world production in 2019 was 1.6 megatons cultivated on 1.7 mega ha [12]. Buckwheat remains thus a marginal crop compared to cereals. For example, maize (*Zea mays* L.) and wheat (*Triticum* sp. L.) productions were, respectively, 1148 and 766 megatons worldwide in 2019 [12]. In 2018, buckwheat yield ranged from 0.4 tons per ha in South Africa to 3.3 tons per ha in France with a mean world yield of 0.94 tons per ha [12]. Buckwheat species have strong adaptability to adverse environmental conditions and represent a good opportunity for organic farming [3,4,13,14]. In most western European countries, buckwheat production has declined during the 20th century with the development of more productive crops, but it is currently receiving renewed attention for its nutritional and environmentally friendly qualities [4,15,16,17].

Buckwheat is used for food consumption, mainly as flour or groats [9]. It is also used to produce noodles, porridge, bread, pancakes, sprouts for salads and smoothies, and even drinks [18]. Buckwheat is considered to have high nutritional value and medicinal qualities and its benefits have been highlighted in several reviews [2,4,9,10,18,19,20,21,22]. Buckwheat is known to be gluten-free which makes it interesting for people with celiac disease [20]. It contains a well-balanced amino acid composition with high concentrations of lysine and arginine compared to cereals [20,23]. It is also rich in dietary fibers, minerals and vitamins [20]. Finally, buckwheat contains high concentrations of antioxidants such as polyphenols and flavonoids [9]. The potential health benefits of buckwheat consumption include anticancer, anti-inflammatory, hypoglycemic, and hypocholesterolemic activities, which are presumably associated with health-promoting compounds such as proteins and phenolic compounds [9,22,24,25]. These qualities have increased buckwheat demand in recent years and have attracted the attention of food scientists and their research [21,22].

It is well known that environmental conditions and agricultural practices may affect the nutritional qualities of crops [26,27]. In this paper, we compared plant growth, yield-related parameters and seed nutritional qualities of two varieties of *F. esculentum* and *F. tataricum* under field conditions in Belgium. The selected varieties were La Harpe and Darja for *F. esculentum* and Islek and Zlata for *F. tartaricum*. La Harpe is a French variety that was previously grown under field conditions in Belgium [28], and Darja is an important variety cultivated in Slovenia [29]. Islek is a domestic population from the Islek region of Europe (border region of Luxemburg, Germany and Belgium) [21,30], and Zlata is a Slovenian variety [31].

## 2. Results

*Fagopyrum esculentum* var. Darja and var. La Harpe and *Fagopyrum tataricum* var. Islek and var. Zlata were sown under field conditions in Belgium, respectively, on 22 May 2019 and 7 June 2020. Seed density was 37 kg/ha for *F. esculentum* (1.48–1.35 × 10^6^ seeds/ha for var. Darja, 1.23–1.02 × 10^6^ seeds/ha for var. La Harpe in 2019 and 2020, respectively) and 30 kg/ha for *F. tataricum* (1.56–1.55 × 10^6^ seeds/ha for var. Islek and 1.45–1.40 × 10^6^ seeds/ha for var. Zlata in 2019 and 2020, respectively). Harvest took place, respectively, on 17 September 2019 and 11 September 2020. Weather conditions during buckwheat cultivation are presented in Figure S1. In 2019, the mean daily temperature was 18.1 ± 3.6 °C with a minimum temperature of 6.7 °C and a maximum temperature of 39.6 °C. The mean daily rainfall was 1.8 ± 5.8 mm with 69 days without rain and two dry periods (22 June–11 July and 19 August–15 September) and a maximum of 42 mm of rainfall on 27 July. The mean daily irradiance was 1.8 ± 0.5 KWh/m^2^d with a minimum of 0.3 KWh/m^2^d and a maximum of 2.8 KWh/m^2^d and a total irradiance of 177 KWh/m^2^ over the growing season. In 2020, the mean daily temperature was 18.6 ± 3.7 °C with a minimum temperature of 8.7 °C and a maximum temperature of 34.9 °C. The mean daily rainfall was 1.4 ± 3.6 mm with 56 days without rain and one dry period from 8 July to 12 August and a maximum of 27 mm of rainfall on 18 August. A drought period of 22 days without rain occurred before sowing in 2020 (Figure S1). In 2020, the mean daily irradiance was 1.8 ± 0.6 KWh/m^2^d with a minimum of 0.6 KWh/m^2^d and a maximum of 2.9 KWh/m^2^d and a total irradiance of 210 KWh/m^2^ over the growing season. The sum of the daily average temperature (base 8) to harvest was 1374 °C in 2019 and 1199 °C in 2020. The sum of precipitations from sowing to harvest was 214 mm in 2019 and 156 mm in 2020.

### 2.1. Plant Morphology

Both species differed regarding plant architecture and reproductive growth (Table 1). Globally, *F. esculentum* showed a smaller plant height (F_1,24_ = 5.34, *p* = 0.029), produced less nodes on the main stem (F_1,37_ = 46.1, *p* < 0.001) and less branches (F_1,37_ = 12.9, *p* < 0.001) than *F. tataricum.* Regarding reproductive growth, *F. esculentum* flowered before *F. tataricum* (F_1,37_ = 24.7, *p* < 0.001). *Fagopyrum tataricum* produced more inflorescences per plant than *F. esculentum* (F_1,37_ = 24.6, *p* < 0.001) but with fewer flowers per inflorescence (F_1,37_ = 115.23, *p* < 0.001). The seeds of *F. esculentum* were also larger than the ones of *F. tataricum* (F_1,18_ = 23.9, *p* = 0.001).

We also observed strong differences between varieties. In *F. esculentum*, the plants of var. La Harpe were 28% smaller (F_1,10_ = 31.4, *p* < 0.001), produced 48% less leaves (F_1,10_ = 16.2, *p* < 0.001) and 12% less inflorescences (F_1,10_ = 6.4, *p* = 0.021) than plants of var. Darja. However, they produced 46% more flowers (F_1,10_ = 44.9, *p* = 0.003) and five times more seeds (F_1,10_ = 76.5, *p* < 0.001) per inflorescence than var. Darja (Table 1). The seed set was three times higher in var. La Harpe than in var. Darja (F_1,10_ = 76.5, *p* < 0.001) and the thousand-grain weight was 26% higher in the former than in the latter (F_1,4_ = 9.95, *p* = 0.0.34, Table 1). We observed less difference among varieties of *F. tataricum* than of *F. esculentum*. Vegetative growth and reproductive growth parameters were indeed similar in var. Islek and var. Zlata (Table 1) with the exception of the seed weight (F_1,4_ = 12.01, *p* = 0.03) and the seed set (F_1,10_ = 82.3, *p* = 0.009), which were slightly higher in var. Zlata than in var. Islek.

The obtained yield was 2370 kg/ha in var. Darja and 2778 kg/ha in var. La Harpe of *F. esculentum* in 2019, while it was 2444 kg/ha in var. Islek and 3667 kg/ha in var. Zlata of *F. tataricum*. In 2020, the yield was, respectively, 2371 kg/ha, 2037 kg/ha, 2518 kg/ha and 2260 kg/ha in var. Darja, var. La Harpe, var. Islek and var. Zlata. In 2020, the mean thousand grain weight was 25.0 g in var. Darja, 30.4 g in var. La Harpe, 19.4 g in var. Islek and 20.8 g in var. Zlata.

### 2.2. Nutritional Qualities

**Figure 1:**
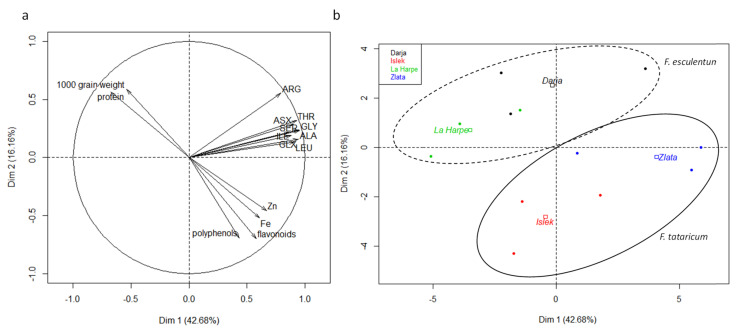
Principal component analysis (PCA) of seed nutritional parameters of *F. esculentum* (var. Darja and var. La Harpe) and *F. tataricum* (var. Islek and var. Zlata) grown under field conditions in Belgium. (**a**) Variable graph of PCA showing nutritional parameter (only parameters with cos^2^ > 0.6 were shown). (**b**) Individual graph showing the varieties of *F. esculentum* and *F. tataricum*.

A principal component analysis (PCA) was performed in order to identify potential differences between species and varieties regarding nutritional qualities of seeds (Figure 1). The PCA showed that 42% of the variance was explained by axis 1 (Dim1) and 16% by axis 2 (Dim2). Axis 1 was mainly explained by, on one side, the protein content, and on the other side, the amino acid, Zn and Fe and flavonoid concentrations (Figure 1a). Axis 2 was mainly explained by, on one side, the seed weight, and on the other side, the total flavonoid and polyphenol concentrations (Figure 1a). Figure 1b showed a clear separation between varieties and species.

The protein content was higher in *F. esculentum* than in *F. tataricum* (F_1,10_ = 16.7, *p* = 0.002) while the total amino acid concentration was similar in both species (Table 2). The essential amino acid percentage was around 31% and 28%, respectively, in *F. esculentum* and *F. tataricum*. It has to be noticed that tryptophan, which is an essential amino acid, could not be quantified with the used technique. The main amino acids were glutamine + glutamic acid and arginine in both species. The amino acid profile did not differ between species, with the exception of tyrosine, which was more present in *F. tataricum* than in *F. esculentum* (F_1,10_ = 6.35, *p* = 0.030). Regarding differences among varieties, methionine concentration was higher in var. Zlata than in var. Islek in *F. tataricum* (F_1,4_ = 22.23, *p* = 0.009, Table 2). The concentrations of alanine (F_3,8_ = 3.95, *p* = 0.049) and leucine (F_3.8_ = 4.24, *p* = 0.045) were higher in var. Zlata than in var. La Harpe (Table 2).

Regarding mineral content, the seeds of *F. esculentum* contained more Mg (F_1,18_ = 7.83, *p* = 0.005) and less Na (F_1,18_ = 7.26, *p* = 0.0158), Fe (F_1,18_ = 17.26, *p* = 0.0007) and Zn (F_1,18_ = 7.88, *p* = 0.0116) than the ones of *F. tataricum*, but the concentrations of K, Ca and Cu were similar whatever the species (Table 3). The highest concentration of Na was observed in var. Islek and the lowest in var. La Harpe, while the highest concentrations of Fe and Zn was observed in var. Zlata and the lowest in var. Darja. Seeds of var. La Harpe were richer in Mg than in the other varieties (F_3,16_ = 8.15, *p* = 0.002).

The main difference between buckwheat species regarding seed quality concerned the antioxidant content (Table 4). The total concentrations of polyphenols (F_1,10_ = 7.41, *p* = 0.006) and flavonoids (F_1,10_ = 8.3, *p* = 0.004) were, respectively, 1.5 and 60 times higher in *F. tataricum* than in *F. esculentum*. Although there was no difference between varieties of a same species for the total polyphenol content, the total flavonoids content was higher in the seeds of var. Darja than in the ones of var. La Harpe in *F. esculentum* (F_1,40_ = 11.7, *p* = 0.026).

## 3. Discussion

Two varieties of *F. esculentum* and of *F. tataricum* were compared under field conditions and the nutritional qualities of the harvested seeds were analyzed. Both species differed in their architecture mainly regarding the number of branches, the number of inflorescences per plant, the number of flowers per inflorescence and the seed weight. *Fagopyrum tataricum* was described as smaller and slenderer with seeds 40% smaller than *F. esculentum* [3]. The main differences between the buckwheat species are related to plant reproduction. *Fagopyrum esculentum* is self-incompatible, distylous and depends on insect pollination for fertilization and seed production [32,33]. *Fagopyrum tataricum* produces smaller flowers that are homostylous and self-compatible and does not depend on insect pollination to produce seeds [33]. Such difference may explain the higher seed set and yield observed in *F. tataricum* than in *F. esculentum*. It was previously observed that the yield of *F. tataricum* was higher than in *F. esculentum* and it was suggested to be explained by the selfing of *F. tataricum* [21]. In our growing conditions, the yield ranged between 2037 kg/ha and 3667 kg/ha depending on the species and year. In previous works in the same region, yields ranged between 2000 and 2500 kg/ha for *F. esculentum* var. La Harpe [28], which are in agreement with our results. These yields are higher than the worldwide and European average over the last 5 years for *F. esculentum* that were, respectively, 1034 kg/ha and 1068 kg/ha [12]. The average buckwheat yield of the European Union over the last 5 years was about 1797 kg/ha and ranged from 3411 kg/ha in France to 798 kg/ha in Estonia [12]. The yields we obtained for *F. esculentum* in our study were thus similar to the most productive European countries. Although the observed yields were lower than those obtained in France [12], they were similar to those obtained in Czech Republic [12] or Serbia [34]. In western Europe, buckwheat is often cultivated in poor soils or marginal area since it can grow under a variety of climatic conditions in a wide range of soils [15,17,35]. In our experiment, buckwheat was cultivated in loamy soil with high yield potential, which could partly explain the high yields we obtained. We observed that the yield was maintained despite the hot and dry periods that occurred in summer in Belgium over these last few years. However, meteorological parameters were shown to significantly affect buckwheat yields and drought and high temperature could decrease average yield [15,36]. Although both species could be affected by drought and heat stress mainly regarding reproductive growth, it was shown that they develop resistance mechanisms and that *F. tataricum* was less affected by water stress than *F. esculentum* [13,14]. Under stressful periods, the long-lasting flowering of buckwheat may be an advantage allowing compensation to maintain yield.

Buckwheat seeds are known to be rich in high quality carbohydrates, protein and amino acid, fatty acid, vitamins, minerals and bioactive compounds such as polyphenols [22]. However, the total content of components depends on variety or environmental factors [2,15,36]. We observed that both species differed by their protein and mineral content in the seeds (Figure 1). Seeds of *F. esculentum* contained more proteins than seeds of *F. tataricum* although their amino acid profile was similar at the exception of tyrosine. Seeds of *F. esculentum* contained also more Mg and less Fe and Zn than *F. tataricum* and flavonoid content was higher in the latter. Moreover, differences were observed between varieties regarding nutritive compound concentrations.

The total protein content we observed was similar to what was previously recorded in buckwheat and was higher than the protein content of cereals such as rice or maize [2,22]. It was reported that the protein content in *F. esculentum* and *F. tataricum* ranged from 6.4% to 18.9% depending on the species and environment [2,22]. The protein content of the seeds cultivated in Belgium was thus quite high as it ranged between 12.8 and 16.1% in our experiment. When expressed as total protein produced per ha, we observed that var. Darja produced 346 kg protein/ha, var. La Harpe produced 447 kg protein/ha, var. Islek produced 312 kg protein/ha, and var. Zlata produced 469 kg protein/ha in 2019. The protein content also depends on the seed fraction: hull only contains 4% proteins while embryon contains 56% and buckwheat flour contains from 8.5% to 19% depending on the variety and growing conditions [2]. The protein content in flour of *F. esculentum* and *F. tataricum* was reported to be similar although it may differ among varieties [37]. Buckwheat proteins have high biological values although their digestibility is relatively low [18,37]. Regarding amino acid, buckwheat is mainly known for its balanced amino acid composition and its high level of lysine and arginine compared to cereals [2,11,18,22]. These amino acids are indeed generally limiting in cereals [11]. Arginine was the second most important amino acid detected in our experiment. The proportion of essential amino acid was around 30% in our experiment. The concentration of essential amino acid was similar between varieties at the exception of the concentration of methionine and leucine that did not differ between species but well among varieties. It was indeed reported that amino acid composition of *F. esculentum* and *F. tataricum* was rather similar [18,21], as observed in our study. Ge and Wang [38] observed low ratio of lysine/arginine (0.79) and methionine/glycine (0.22) in Tartary buckwheat, arguing that these ratios are critical factors that determine the cholesterol-lowering effects of plant proteins and that the lower they are the better the cholesterol-lowering effects [11,38]. These ratios are indeed lower in buckwheat than in most plants [11]. In our experiment, we observed lysine/arginine ratios ranging between 0.27 and 0.36 and methionine/glycine ratios ranging from 0.14 to 0.28 depending on the variety. These results suggest a potential cholesterol lowering effect in both species.

Mineral elements are very abundant in buckwheat, particularly K, P, Cu, Ca, Se, Mg, Ba, B, I, Fe, Pt, Zn and Co [22]. Buckwheat contains more K, Mg, P, Ca, Fe, Zn, Cr, Cu and Mn than the main cereals [11,22]. Buckwheat could thus be an important source of microelements such as Fe, Mn and Zn [2]. Moreover, the bioavailability of Zn, Cu and K from buckwheat is high compared to other crops [11]. We confirmed the high content of K and Fe in the seeds in our growing conditions. The seed mineral content observed in our growing conditions falls into the range of other studies for *F. esculentum* and *F. tataricum* [21,22]. It has to be noticed that mineral distribution in the seed depends on the tissues [21,22] and mineral proportion ranges from 2.0 to 2.5% in the whole grains, 1.8 to 2.0% in kernel, 2.2 to 3.5% in dehulled grains, 0.8 to 9% in flour and 3.4 to 4.2% in hulls [2,22]. We analyzed the minerals in the whole grains in this study. It was reported that P, K and Mg are most concentrated in bran [2] and trace elements were excessively present in the outer membrane of seeds and seed coat [22]. However, Pongrac et al. showed that valuable essential elements such as Mg, P, S, K, Mn, Fe and Zn are located in the embryo [18,39]. We also observed differences between buckwheat species regarding the concentration of Mg, Na, Fe and Zn. A higher proportion of Fe and Zn in *F. tataricum* compared to *F. esculentum* was previously reported [21,22]. Moreover, mineral content may vary among varieties inside a same species as previously reported [21].

Seeds of both species contain polyphenols, and seeds of *F. tataricum* were particularly rich in flavonoids compared to *F. esculentum*. Such difference between species regarding total flavonoids content was not observed in other organs such as leaves and inflorescences in the same varieties [13,14]. The taste of *F. tataricum* seeds is more bitter than that of *F. esculentum* due to the higher concentrations of flavonoids [21]. Flavonoids are the prominent group of polyphenol secondary metabolites and rutin is the main flavonoid observed in buckwheat as it accounts for 90% of the total phenolics [22]. The content of polyphenols and flavonoids depends on various factors including plant growth stage, organ, species or growing season [22]. The content of flavonoids and of rutin in particular was reported to be usually higher in *F. tataricum* than in *F. esculentum* [21,22,37]. However, it varied among varieties in the same species [37]. Rutin content is lower in seeds of *F. esculentum* than in the other organs, and seeds of *F. tataricum* contain up to 70 times more rutin than the ones of *F. esculentum* [21,22]. On average, flour of *F. tataricum* contains 10 times more total flavonoid content than *F. esculentum* [21]. Rutin tends to prevent flour deterioration in buckwheat [18]. It is known that rutin content in the seed decreases during seed ripening and increases during seed germination so that rutin content in sprouts is significantly higher than in ungerminated seeds [22]. Buckwheat is the only grain crop with rutin content that is known to have antioxidant, anti-inflammatory, and anti-carcinogenic properties [11]. In addition to rutin, more than 130 major polyphenols have been isolated in buckwheat [21,22].

## 4. Materials and Methods

### 4.1. Plant Material and Growth Conditions

Seeds of *Fagopyrum esculentum* var. Darja and *Fagopyrum tataricum* var. Zlata were obtained from Prof. Dr. Ivan Kreft (University of Ljublana, Slovenia). Seeds of *F. tataricum* var. Islek were obtained from Christian Zewen (Luxemburg). Finally, seeds of *F. esculentum* var. La Harpe were purchased at GIRED (Le Thor, France).

Seeds were sowed at the experimental farm of the university (Corroy le Grand, Belgium, 50°40′0.6″ N 4°38′39″ E) on 22 May 2019 and 7 June 2020 at a density of 37 kg/ha for *F. esculentum* and of 30 kg/ha for *F. tataricum.* It corresponds to a seed density of 1.48–1.35 × 10^6^/ha for var. Darja, 1.23–1.02 × 10^6^/ha for var. La Harpe, 1.56–1.55 × 10^6^/ha for var. Islek and 1.45–1.40 × 10^6^/ha for var. Zlata in 2019 and 2020, respectively. Each variety occupied three lines of 6 × 1.5 m organized in a split-plot design. Harvest occurred on 17 September in 2019 and 11 September in 2020. No fertilizers (N, P and K) and pesticides were applied during the cultivation. Buckwheat was cultivated on a loamy soil, which characterized the region. A green manure (*Avena strigosa*) was cultivated before our field trial in 2019, and the buckwheat trials of 2019 and 2020 were performed at the same place with no intercrop between the trials. Fertilization (700 kg/ha, 77 units of P_2_O_5_ and 77 units of K_2_O) was provided before the sowing, respectively, on 11 April 2019 and 5 June 2020. The weather conditions (temperatures, rain, light irradiance, relative humidity) were measured daily at the weather station of the experimental farm of the university.

### 4.2. Plant Description

Two weeks before harvest, morphological observations were performed according to the Buckwheat Descriptor of IPGRI (International Plant Genetic Resources Institute) [40] on 10 plants per variety in 2019: plant height, number of nodes on the main stem, number of branches on the main stem, number of leaves per plant, number of inflorescences per plant, node of the first inflorescence, number of flowers per inflorescence, number of viable and aborted seeds per inflorescence. The aerial parts of five plants per variety were harvested and separated in stems, leaves and inflorescences and dry weights (DWs) were measured after 72 h oven drying at 70 °C. At harvest, yield parameters and thousand-grain weight were analyzed in 2019 and 2020.

### 4.3. Nutritional Qualities of the Seeds

Three replicates of 100 seeds per variety were crushed in liquid nitrogen to quantify mineral (Na, Ca, K, Mg, Cu, Zn, Fe) concentrations, protein content, amino acid composition and total polyphenols and flavonoids concentrations on the harvest of 2019.

For mineral content, seeds were digested with 4 mL HNO_3_ 68% at 80 °C. After complete evaporation, residues were dissolved with HNO_3_ 68% + HClcc (1:3, v:v) and filtered on Whatman Grade A filter before quantification by flame atomic absorption spectrophotometry (ICE 3300, Thermo Scientific, Waltham, MA, USA) using standards (Spectracer-CPACHEM; accredited through ISO/IEC17025).

Total protein content was determined by analyzing the total nitrogen matter according to the Kjeldhal method [41] from 1 g of seed samples in triplicates. Amino acid profile and concentrations were determined according to Meussen et al. [42] from 200 mg of grounded seeds in triplicates after acid hydrolysis and derivatization using phthaldialdehyde (OPA) reagent in combination with 9-fluorenylmethyl chloroformate (FMOC). Samples were exposed to 500 µL of 6M hydrochloric acid containing 1% (*w/v*) of phenol and incubated during 18 h at 110 °C. After drying under vacuum, samples were resuspended in 400 µL methanol + 500 µL MilliQwater. A double derivatization process was performed in pre-columns using (i) 2-mercaptoethanol 4% + 25 mg OPA dissolved in 0.5 mL methanol in a total volume of 5 mL borate buffer pH 10.4 and (ii) FMOC 0.25% in acetonitrile. Samples were injected on a Zorbax Eclipse Plus column (Agilent; 3.5 µm particle size; 150 × 21 mm) maintained at 40 °C. The mobile phase was composed of (A) phosphate buffer 40 mM pH 8.4 and (B) acetonitrile/methanol/water (45:45:10 *v/v/v*)) at a flow rate of 0.42 mL min^−1^ (100% A–0% B 0.5 min; progressive increase from 0 to 57% B 0.5–25 min). OPA-derivatized and FMOC-derivatized amino acids were, respectively, detected at 350 nm and 260 nm excitation and 450 nm and 325 emission wavelengths. Bovine serum albumin (BSA: 1 mg mL^−1^) was used as standard.

Total polyphenols and flavonoids concentrations were quantified as previously described [13] from 100 mg of grounded seeds in triplicates after extraction in 80% methanol. Total phenolic content was determined using the modified Folin–Ciocalteu colorimetric method and absorbances were measured at 760 nm using a spectrophotometer (UV1800, Shimadzu, s-Hertogenbosh, the Netherlands) and a standard curve ranging from 0.0 to 800.0 μg of gallic acid mL^−1^. Total flavonoid content was determined by using the aluminum chloride chelation method, and absorbances were measured at 440 nm using a spectrophotometer (UV1800, Shimadzu, s-Hertogenbosh, the Netherlands) and a standard curve ranging from 0.0 to 50.0 μg of quercetin mL^−1^.

### 4.4. Statistical Analyses

Analyses were conducted in R studio or SAS Enterprise Guide 8.3. The normality of the data was estimated using Shapiro–Wilk tests, and homoscedasticity was verified using Levene’s tests. Morphological parameters were compared between species and varieties using a mixed linear model with the species or variety as fixed factor and the plot as random factor. Nutritional parameters were compared between species and varieties using one-way analysis of variance with the species or variety as fixed factor. Post-hoc comparison between varieties were performed using Tukey test. Principal component analysis was performed to visualize the differences in seed nutritional quality according to the varieties. If not indicated otherwise, data are presented as means ± SD.

## 5. Conclusions

Common and Tartary buckwheat were grown in Belgium under field conditions and compared regarding growth parameters and seed nutritional qualities. Both species differed by their architecture mainly regarding the number of branches, the number of inflorescences per plant, the number of flowers per inflorescence and the seed weight. High yields were obtained for both species compared to other European countries despite the dry and hot periods observed over these last few years during spring and summer. We confirmed the nutritional qualities of the seeds regarding protein, amino acid, mineral content and flavonoids content. Both species differed by their seed quality, mainly in relation to the total protein content, which was higher in *F. esculentum*, and flavonoid, Zn and Fe contents, which were higher in *F. tataricum*.

## Figures and Tables

**Table 1:** Vegetative, reproductive and yield-related parameters of *F. esculentum* and *F. tataricum* under field conditions in 2019.

		*F. esculentum*		*F. tataricum*	
Parameter	n	Darja	La Harpe	Islek	Zlata
Vegetative Growth					
Plant height (cm)	10	137.6 ± 10.2 a	98 ± 13.1 b	126 ± 16.8 a	139.1 ± 18.6 a
Number of leaves	10	58.7 ± 27.2 a	30.5 ± 5.6 b	36.4 ± 25.9 ab	50.7 ± 33.3 ab
Number of branches	10	5.2 ± 0.8 b	4.4 ± 1 b	6.7 ± 1 a	5.5 ± 1.3 ab
Number of nodes	10	15 ± 2.3 b	10.8 ± 3.4 c	20.1 ± 2.3 a	20.4 ± 3.4 a
Stem dry weight (g)	5	7.1 ± 2.7 a	6.5 ± 1.9 a	4.8 ± 1.4 a	5.3 ± 1.4 a
Leaves dry weight (g)	5	0.9 ± 0.3 a	1.2 ± 0.4 a	0.3 ± 0.2 a	0.8 ± 0.8 a
Reproductive Growth					
Node of the first inflorescence	10	6.3 ± 0.8 bc	5.5 ± 1.2 c	7.9 ± 1 a	7.2 ± 0.8 ab
Number of inflorescences	10	69.1 ± 18 ab	45.2 ± 21.1 b	89 ± 40.5 ab	107.6 ± 63.3 a
Inflorescence dry weight (g)	5	1.6 ± 0.8 b	18.1 ± 5.2 a	8.4 ± 3 ab	6.2 ± 1.5 ab
Flowers per inflorescence	10	73.7 ± 20.2 b	108.5 ± 27 a	29.3 ± 5.5 c	31.1 ± 5.8 c
Viable seeds per inflorescence	10	7.5 ± 2.2 c	35.8 ± 10.4 a	11.5 ± 3.1 bc	17 ± 3.9 b
Aborted seeds per inflorescence	10	3.3 ± 2.6 b	15 ± 10.2 a	4.6 ± 2.7 b	3.4 ± 2 b
Seed set (%)	10	10.8 ± 3.3 c	33.3 ± 5.4 b	38.8 ± 4.5 b	51.5 ± 5 a
Thousand-grain weight (g)	5	27.5 ± 0.4 b	36.1 ± 4.7 a	19.3 ± 0.7 c	21.4 ± 0.8 bc

**Table 2:** Protein and amino acid concentrations in seeds of *F. esculentum* and *F. tataricum*.

		*F. esculentum*		*F. tataricum*	
Parameter	n	Darja	La Harpe	Islek	Zlata
Protein content (%)	3	14.6 ± 1.3 ab	16.1 ± 1.2a	12.8 ± 0.1b	12.8 ± 0.9 b
Amino acid concentrations (mg/g FW)					
Asparagine ^1^	3	7.87 ± 0.37 a	7.03 ± 0.51 a	7.47 ± 0.50 a	8.17 ± 0.32 a
Cysteine	3	2.00 ± 0.61 a	1.97 ± 0.15 a	2.10 ± 0.10 a	2.40 ± 0.26 a
Glutamine ^2^	3	18.41 ± 1.82 a	16.62 ± 1.36 a	17.52 ± 1.20 a	20.18 ± 0.90 a
Serine	3	4.88 ± 0.36 a	4.67 ± 0.14 a	4.77 ± 0.13 a	4.95 ± 0.31 a
Histidine ^3^	3	2.22 ± 0.16 a	2.17 ± 0.14 a	2.05 ± 0.10 a	2.17 ± 0.15 a
Glycine	3	6.90 ± 0.49 a	6.37 ± 0.33 a	6.60 ± 0.30 a	7.23 ± 0.42 a
Threonine ^3^	3	4.42 ± 0.33 a	4.00 ± 0.28 a	4.13 ± 0.19 a	4.58 ± 0.50 a
Arginine	3	10.37 ± 0.90 a	9.48 ± 0.36 a	9.25 ± 0.31 a	10.48 ± 0.50 a
Methionine ^3,4^	3	1.60 ± 0.46 ab	1.47 ± 0.42 ab	0.90 ± 0.35 b	2.03 ± 0.23 a
Alanine	3	5.22 ± 0.36 ab	4.82 ± 0.20 b	5.02 ± 0.24 ab	5.53 ± 0.25 a
Tyrosine	3	1.77 ± 0.12 b	1.87 ± 0.23 b	2.07 ± 0.15 a	2.00 ± 0.10 a
Valine ^3^	3	3.20 ± 0.26 a	2.92 ± 0.38 a	3.45 ± 0.13 a	3.18 ± 0.16 a
Phenylalanine ^3^		3.8 ± 0.21 a	3.5 ± 0.21 a	ND	ND
Isoleucine ^3^	3	2.68 ± 0.17 a	2.55 ± 0.73 a	2.63 ± 0.10 a	2.83 ± 0.13 a
Leucine ^3^	3	5.85 ± 0.35 ab	5.37 ± 0.37 b	5.72 ± 0.32 ab	6.4 ± 0.33 a
Lysine ^3^	3	2.87 ± 0.24 a	3.00 ± 0.40 a	2.83 ± 0.16 a	3.80 ± 0.99 a
Proline	3	2.30 ± 0.26 a	1.73 ± 0.15 a	1.93 ± 0.11 a	2.40 ± 0.66 a

^1^ Asparagine + aspartic acid; ^2^ glutamine + glutamic acid; ^3^ essential amino acids; ^4^ methionine sulfone; ND: no data; FW: fresh weight.

**Table 3:** Mineral concentrations in seeds of *F. esculentum* and *F. tataricum*.

		*F. esculentum*		*F. tataricum*	
Mineral	n	Darja	La Harpe	Islek	Zlata
K (mg/g DW)	5	3.72 ± 0.48 a	4.58 ± 0.24 a	4.16 ± 0.13 a	4.37 ± 0.90 a
Na (mg/g DW)	5	1.86 ± 0.65 ab	1.10 ± 0.32 b	2.54 ± 1.08 a	2.36 ± 0.60 ab
Ca (mg/g DW)	5	0.16 ± 0.09 a	0.22 ± 0.07 a	0.21 ± 0.04 a	0.11 ± 0.04 a
Mg (mg/g DW)	5	1.22 ± 0.17 b	1.50 ± 0.12 a	1.17 ± 0.07 b	1.13 ± 0.08 b
Cu (µg/g DW)	5	6.57 ± 1.74 a	5.82 ± 0.95 a	6.75 ± 0.60 a	5.88 ± 1.38 a
Fe (µg/g DW)^3^	5	19.90 ± 2.33 b	25.94 ± 3.05 ab	30.51 ± 4.21 ab	34.66 ± 5.01 a
Zn (µg/g DW)	5	18.14 ± 3.06 b	21.17 ± 2.80 ab	22.85 ± 1.88 ab	26.17 ± 4.80 a

DW: dry weight.

**Table 4:** Antioxidant concentrations in seeds of *F. esculentum* and *F. tataricum*.

		*F. esculentum*		*F. tataricum*	
Antioxidant	n	Darja	La Harpe	Islek	Zlata
Flavonoids (mg/g FW)	3	0.10 ± 0.03 b	0.02 ± 0.01 b	3,44 ± 103 a	3,79 ± 105 a
Polyphenols (mg/g FW)	3	3.00 ± 0.13 b	3.25 ± 0.17 ab	4.48 ± 1.07 ab	4.90 ± 0.34 a

FW: fresh weight.

## Data Availability

The data presented in this study are available in the text and supplemental data. The data presented in this study are available on request from the corresponding author.

## Supplementary Materials

The following are available online at https://www.mdpi.com/2223-7747/10/2/258/s1, Figure S1: Weather conditions in the field in 2019 and 2020 during buckwheat culture in Corroy-le-Grand, Belgium. (a,b) Temperature and precipitations and (c,d) light irradiance on (a,c) 2019 and (c,d) 2020. Sowing and harvest dates are indicated by red vertical bars.
