# Supplementary material for: Comparison of Plant Morphology, Yield and Nutritional Quality of Fagopyrum esculentum and Fagopyrum tataricum Grown under Field Conditions in Belgium
Source: Plants (Basel). 2021 Jan 28;10(2):258. doi: 10.3390/plants10020258 (PMC7910852; doi:10.3390/plants10020258)
Supplement: Supplementary file 1 [file plants-10-00258-s001.pdf]

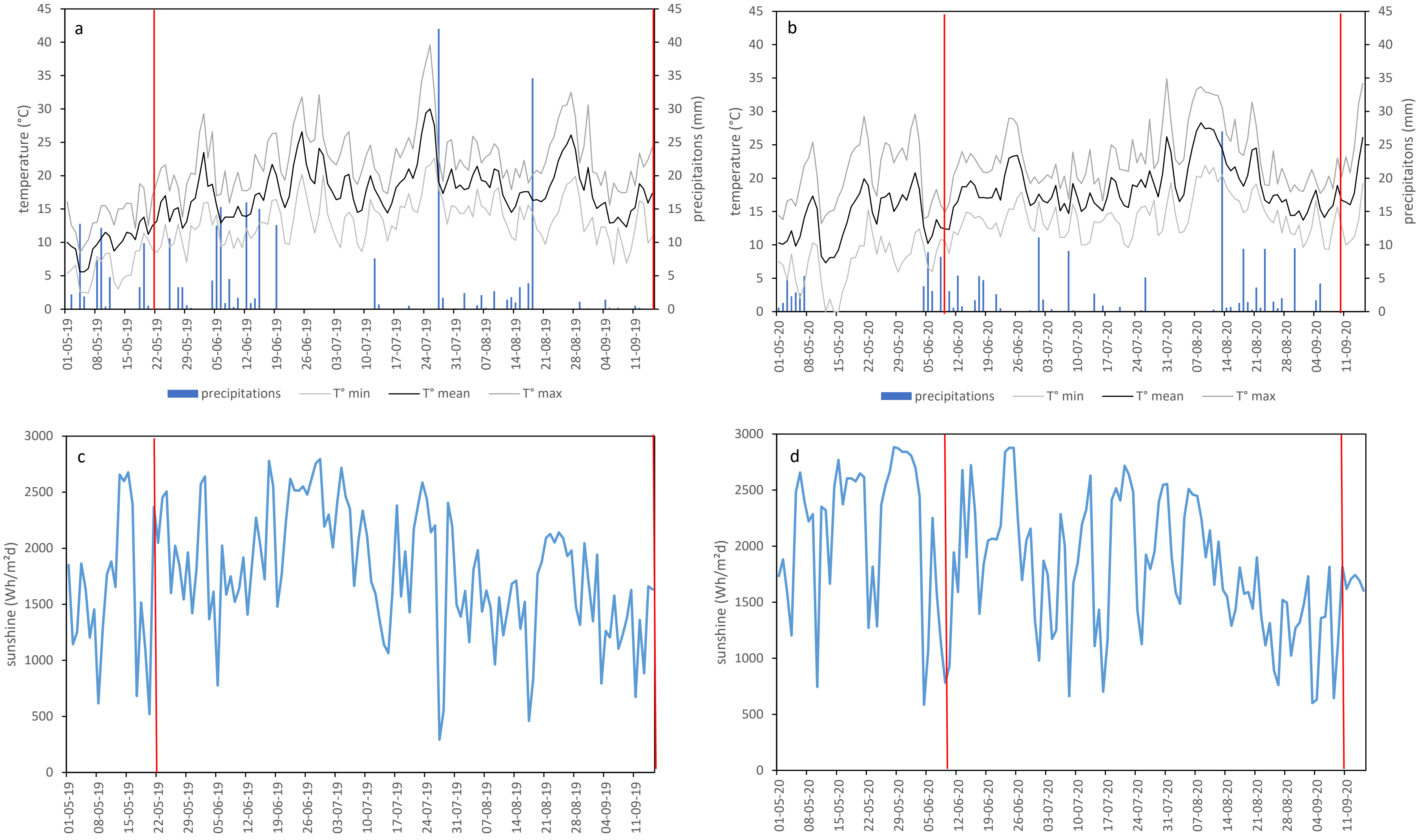

Figure S1 : Weather conditions in the field in 2019 and 2020 during buckwheat culture in Corroy-le-Grand, Belgium. (a, b) temperature and precipitations and (c, d) light irradiance on (a, c) 2019 and (c, d) 2020. Sowing and harvest dates are indicated by red vertical bars.
